# Hyperhomocysteinemia accompany with metabolic syndrome increase the risk of left ventricular hypertrophy in rural Chinese

**DOI:** 10.1186/s12872-020-01350-2

**Published:** 2020-02-03

**Authors:** Shasha Yu, Yintao Chen, Hongmei Yang, Xiaofan Guo, Liqiang Zheng, Yingxian Sun

**Affiliations:** 1 Department of Cardiology, The First Hospital of China Medical University, 155 Nanjing North Street, Heping District, Shenyang, 110001 China; 2 Department of Clinical Epidemiology, Shengjing Hospital of China Medical University, Shenyang, 117004 China

**Keywords:** Hyperhomocysteinemia, Metabolic syndrome, Left ventricular hypertrophy, Predictor, Rural

## Abstract

**Background:**

To investigate the influence of hyperhomocysteinemia (HHcy) and metabolic syndrome (MetS) on left ventricular hypertrophy (LVH) in residents in rural Northeast China.

**Methods:**

We performed a cross-sectional baseline data analysis of 6837 subjects (mean age: 54 ± 10 years) recruited from a rural area of China. Anthropometric indicators were measured according to standard methods. MetS was defined by the modified ATP III criteria. HHcy was defined according to the WHO standard: an Hcy level > 15 μmol/L representing HHcy. Four groups were defined: non-HHcy & non-MetS, HHcy & non-MetS, MetS & non-HHcy and HHcy & MetS.

**Results:**

The left ventricular mass index for height^2.7^ (LVMH^2.7^) in both sexes was significantly higher in the HHcy & MetS group than in the non-HHcy & non-MetS group (females: 51.23 ± 16.34 vs. 40.09 ± 10.55 g^-2.7^, *P* < 0.001; males: 48.67 ± 12.24 g^-2.7^ vs. 42.42 ± 11.38 g^-2.7^, *P* < 0.001). A similar result was observed in those groups when using the left ventricular mass index (LVMI) for body surface area to define LVH (females: 103.58 ± 31.92 g^− 2^ vs. 86.63 ± 20.47 g^− 2^, *P* < 0.001; males: 106.10 ± 24.69 g^− 2^ vs. 98.16 ± 23.29 g^− 2^, *P* < 0.001). The results of multiple regression analysis indicated that the HHcy & MetS group had a higher risk of LVH than the other three groups (OR: 1.628 for LVMI, *P* < 0.001, OR: 2.433 for LVMH^2.7^, *P* < 0.001). Moreover, subjects in the HHcy & non-MetS group [OR (95% CI): 1.297 (1.058, 1.591) for LVMI, *P* < 0.05; OR (95% CI): 1.248 (1.044, 1.492) for LVMH^2.7^, *P* < 0.05] also had a statistically greater risk of LVH than subjects in the non-HHcy & non-MetS group. The HHcy & non-MetS group was also found to be significantly and independently associated with LVH.

**Conclusion:**

Hyperhomocysteinemia has an independent effect on LVH. The combined effect of MetS and hyperhomocysteinemia might increase the strength of the abovementioned effects.

## Background

Hyperhomocysteinemia has been reported to be relevant in cardiovascular diseases linked to atherosclerosis and is considered an independent marker of many cardiovascular risk factors [1]. Zhao and colleagues claimed that the combination of hyperhomocysteinemia and hyperuricemia could result in accelerated atherosclerosis [2]. In addition, Zhang and colleagues confirmed that the coexistence of hyper-homocysteinemia and elevated blood pressure increased the risk of early atherosclerosis in the carotid artery [3]. The possible mechanisms may include a change in the total antioxidant status and regeneration of endothelial cells or a decrease in the synthesis of high-density lipoprotein [4].

LVH has already been confirmed to be independently correlated with the deterioration of health and increased risk of cardiovascular death. Additionally, LVH statistically increases the risks of myocardial infarction, dysfunctions of the heart, stroke and sudden cardiac death [5]. One study claimed that homocysteine levels are explicitly associated with cardiac systolic function in subjects with coronary artery disease (CAD) [6]. Furthermore, Nesrin and Sarıman et al. reported that, in all obstructive sleep apnea syndrome (OSAS) groups, the homocysteine levels were elevated and accompanied by echocardiographic changes, such as left ventricular (LV) hypertrophy and cardiac diastolic dysfunction [7]. All of the previous studies that aimed to confirm the association between left ventricular hypertrophy and homocysteine were conducted in patients with specific clinical diseases. There is a lack of research on the possible link between homocysteine and left ventricular hypertrophy among the general population. It is important to determine this relationship in the general population with various backgrounds in terms of genetic predisposition, food customs, and environments, which are different from those of previous studies. In rural China, the prevalence of MetS is 39.0% [8]. The relationship between MetS and LVH has previously been reported [9, 10]. An epidemiological study named PAMELA (Pressioni Arteriose Monitorate E Loro Associazioni) confirmed that an elevation of the left ventricular mass index and an increased rate of LVH are the major characteristics of MetS-associated heart problems. Metabolic syndrome markedly increases the risk of cardiovascular disease and all causes of death (71 and 37%, respectively) [11]. However, it is still unknown whether the coexistence of HHcy and MetS has a worse deteriorative effect on cardiac remodeling. We conducted research in rural Northeast China and enrolled the general population to estimate whether HHcy is linked to LVH and whether HHcy combined with the presence of MetS increases the risk of LVH.

## Methods

### Study population

In our previous paper, we described the characteristics of the study in detail [8]. From January 2001 to August 2003, we enrolled residents older than 35 years to evaluate the prevalence, morbidity rate and historical process of CVD in villages in Liaoning Province in the Northeast area of China. The exclusion criteria were previously described [12].

Pregnant subjects and those who had a mental disorder or malignant tumors and had severe psychiatric disturbances, hepatic failure, or end-stage renal failure were excluded in the present research. We asked 14,016 eligible residents from different villages who were 35 years or older to participate in our research. Of the 14,016 residents that were asked, 85.3% responded and agreed to participate and complete the research. This research was approved by the Ethics Committee of the Chinese Medical University (China Shen Yang, AF-SDP-07-1,0–01). The research procedures followed the ethically normative criteria. Participants’ welfare, medical plans and confidentiality agreements associated with their contact details were determined before the research began. Then, a written consent form was given to the participants. In this report, we used baseline data, and only participants with a complete set of data regarding the variables to be analyzed in the study were included, making the final sample size 6837 (3150 men and 3687 women). In terms of the possible disorders that were responsible for left ventricular hypertrophy, 1451 had hypertension, 320 had angina pectoris and 98 had myocardial infarction, 52 had atrial fibrillation and 69 had heart failure.

### Data collection and measurements

#### Data collection

All of the data in our study were gathered by a skilled, trained cardiologist and nurses during clinical face to face interviews with a standardized questionnaire. We conducted an organized training session for all of the investigators before the study to ensure that they were qualified to participate in the interview [8, 12]. After the training session, the participants took a test to determine whether they were qualified to collect data. Further guidance and assistance were provided during the investigation. The data obtained through the interviews with the standard questionnaire included current drinking status, current smoking status, exercise status and educational level [8, 12]. The central steering committee and quality control committee were responsible for guiding this study. Exercise status was categorized into the following three levels: low, moderate and high, and the specific standard was previously described [8]. Education levels were divided into primary school or below, middle school and high school.

#### Blood pressure measurement

For the blood pressure (BP) measurement, we used a standard protocol, as many guidelines have recommended. The protocol stated that the participants should be at rest for at least five minutes and not consume caffeinated drinks or exercise before the BP measurement. Omron Healthcare automatic electronic sphygmomanometers (HEM-907; Omron Healthcare, Kyoto, Japan) were used to measure participants’ BP. We used the average of three BP measurements in all analyses.

#### Waist circumference measurement and body mass index calculation

While measuring height and weight, the study subjects were asked to wear light clothes and remove their shoes. We recorded participants’ height and weight to an accuracy of 0.1 cm and 0.1 kg. Subjects’ waist circumferences (WC) were also measured with nonelastic tape at the umbilicus (0.1 cm). Body mass index (BMI) was calculated as body weight (kg)/ height (m) ^2^.

#### Blood sample and biochemistry test

Participants were required to fast for at least twelve hours, and fasting blood specimens were taken in the morning. As previously described, we used enzymatic analysis to exam total cholesterol (TC), triglycerides (TGs), fasting blood glucose (FPG), low-density lipoprotein cholesterol (LDL-C), and high-density lipoprotein cholesterol (HDL-C) with an automatic biochemical analyzer and biochemical indicators [8, 9]. The technician calibrated the laboratory equipment before use, and specimen analyses were repeated using blind specimens.

#### Transthoracic echocardiography evaluation

We recorded M-mode measurements at the end of diastole and the end of systole according to the recommendations of the American Society of Echocardiography (ASE) [13]. The details of the procedures were previously described [8]. The average of five consecutive cardiac cycles was used to calculate the echocardiographic data. A single cardiologist read the images without knowing the subjects’ clinical characteristics.

#### Definitions

Currently, there is no uniform definition of HHcy. According to the WHO standard, the average level of Hcy for healthy adults is 5–15 μmol/L, with an Hcy level > 15 μmol/L representing HHcy [14]. MetS is diagnosed according to the modified NCEP ATP III criteria [15]. At least 3 or more of the following 5 components are needed to diagnose MetS:

**Table:** 

Components	Criteria
Elevated WC	≥ 102 cm (Male); ≥ 88 cm (Female)
Elevated TG	> 150 mg/dL (1.7 mmol/L)
Reduced HDL-C	< 40 mg/dL or 1.04 mmol/L (Male) < 50 mg/dL or 1.29 mmol/L (Female)
Hypertension or elevated BP	≥130/85 mmHg
Diabetes or elevated FPG	≥ 5.6 mmol/L

WC ≥ 88 cm for females and WC ≥ 102 cm for males is defined as abdominal obesity [16].

TC ≥ 6.21 mmol/L (240 mg/dL) means high TC, while high LDL-C is diagnosed as the concentration of LDL-C ≥ 4.16 mmol/L (160 mg/dL). According to the WHO criteria, FPG ≥ 7 mmol/L (126 mg/dL) and/or being treated for diabetes was diagnosed as diabetes [17].

The LVM was calculated according to the formula presented by Devereux and Reichek [18]:

**Table:** 

LVM (g) = 1.04 × [(LV end-diastolic dimension (LVEDD)^*^ + end-diastolic interventricular septum thickness (IVSd)^#^ + end-diastolic LV posterior wall septum thickness (PWd)^$^)^3^-(LVEDD)^3^]-13.6	
^*^ LVEDD is the end-diastolic LV internal diameter; ^#^ IVSd is the ventricular septal thickness; ^$^ PWd is the posterior LV wall thickness	

The LVM was indexed by both body surface area (LVMI) and height raised to a power of 2.7 (LVMH^2.7^), as suggested by De Simone et al. [18].

We used a limit of 51 g^-2.7^ in either sex to separate normal left ventricular thickness from LVH because its value is related to prognostics [19]. High LVMI was defined as LVMI larger than 115 g/m^2^ for male patients and larger 95 g/m^2^ for female patients, as defined by the ASE recommendations. Participants who never smoked or drank were defined as never smokers or never drinkers, and those who were currently smoking and drinking were defined as current smokers or current drinkers. Physical activity was evaluated using questions that have been described in many previous studies and were similar to those used and validated in the “Seven Countries Study” [20].

## Results

The average age of the total eligible participants was 54.42 ± 10.73 years. The proportions of males and females were 46.1 and 53.9%, respectively. Of the 6837 subjects, 696 (10.2%) had diabetes and 3576 (52.3%) had hypertension. The median homocysteine level was 17.34 μmol/L (IQR: 17.05–17.64). The proportion of hyperhomocysteinemia was 41.3% (2821/6837). Among the study subjects, 2660 participants (38.9%) had MetS while 1057 (39.7%) had hyperhomocysteinemia (Table 1).

In Table 1, the characteristics of the participants are shown according to the elevated homocysteine level and MetS. The participants in the non-HHcy coexistent with non-MetS subgroups were younger compared with those in the other groups. The participants with HHcy alone and with HHcy coexistent with MetS were more likely to be men than those in the other group.

**Table 1:** Demographic and clinical data of the study population subdivided according to the presence or the absence of Metabolic syndrome and Hyperhomocysteinemia in rural Northeast China

Variables	Overall (*n* = 6837)	MetS (−) (*n* = 4177)		MetS (+) (*n* = 2660)	
		HHcy (−)0 (*n* = 2413)	HHcy (+)1 (*n* = 1764)	HHcy (−)2 (*n* = 1603)	HHcy (+)3 (*n* = 1057)
Age, years	54.42 ± 10.73	51.25 ± 9.84	56.53 ± 11.69*	54.37 ± 9.70*^#^	58.22 ± 10.40*^#^^
Homocysteine (μmol/L)	17.34 ± 12.37	11.35 ± 2.39	25.63 ± 15.11*	11.34 ± 2.25^#^	26.32 ± 16.01*^^^
Gender, male (%)	3150(46.1)	902(37.4)	1266(71.8)*	381(23.8)*^#^	601(56.9) *^#^^
Diabetes (%)	696(10.2)	82(3.4)	51(2.9)	350(21.8)*^#^	213(20.2)*^#^
Hypertension (%)	3576(52.3)	841(34.9)	834(47.3)*	1095(68.3)*^#^	806(76.3)*^#^^
High TC (%)	874(12.8)	232(9.6)	160(9.1)	273(17.0) *^#^	209(19.8)*^#^^
High TG (%)	1204(17.6)	105(4.4)	85(4.8)	577(36.0)* ^#^	437(41.3)*^#^^
High LDL-C (%)	390(5.7)	93(3.9)	70(4.0)	118(7.4) *^#^	109(10.3)*^# ^^
Low HDL-C (%)	791(11.6)	84(3.5)	96(5.4)*	318(19.8)*^#^	293(27.7)*^# ^^
Hyperuricemia (%)	772(11.3)	116(4.8)	190(10.8)*	197(12.3)*	269(25.4)*^# ^^
Abdominal obesity (%)	1212(17.7)	194(8.0)	88(5.0)*	638(39.8)*^#^	292(27.6)*^# ^^
Body mass index(kg/m^2^)	24.79 ± 3.79	23.51 ± 3.44	23.48 ± 3.30	26.85 ± 3.50*^#^	26.78 ± 3.48*
Waist circumference(cm)	83.52 ± 9.85	79.03 ± 8.52	80.27 ± 8.43*	89.14 ± 8.33*^#^	90.70 ± 8.64*^^^
Fast glucose (mmol/L)	5.87 ± 1.73	5.44 ± 1.17	5.42 ± 0.86	6.59 ± 2.35*^#^	6.48 ± 2.16*
Total cholesterol (mmol/L)	5.08 ± 1.03	4.93 ± 0.95	4.95 ± 0.95	5.29 ± 1.14*^#^	5.31 ± 1.07*
Triglycerides (mmol/L)	1.65 ± 1.61	1.14 ± 1.02	1.17 ± 0.74	2.39 ± 2.20*^#^	2.50 ± 1.90*
HDL cholesterol (mmol/L)	1.44 ± 0.41	1.56 ± 0.38	1.55 ± 0.45	1.26 ± 0.29*^#^	1.24 ± 0.34*
LDL cholesterol (mmol/L)	2.83 ± 0.78	2.71 ± 0.71	2.77 ± 0.73*	2.96 ± 0.83*^#^	3.01 ± 0.84*
Serum creatinine (mmol/L)	70.07 ± 21.65	65.80 ± 11.81	74.84 ± 31.29*	66.34 ± 15.17^#^	77.51 ± 23.96*^^^
Systolic blood pressure (mmHg)	142.91 ± 24.58	133.8 ± 22.22	141.31 ± 24.30*	149.56 ± 22.47*^#^	155.88 ± 24.72*^^^
Diastolic blood pressure (mmHg)	81.73 ± 11.88	77.86 ± 10.63	80.67 ± 11.75*	84.86 ± 11.07*^#^	87.59 ± 12.41*^^^
Current smoker	2547(37.3)	782(32.4)	922(52.3)*	401(25.0)*^#^	442(41.8)*^# ^^
Current drinker	1534(22.4)	506(21.0)	575(32.6)*	198(12.4)*^#^	255(24.1)*^# ^^
Exercised status (%)					< 0.001
Low	1628(23.8)	455(18.9)	419(23.8)	426(26.6)	328(31.0)
Moderate	4772(69.8)	1819(75.4)	1250(70.9)	1043(65.1)	660(62.4)
High	437(6.4)	139(5.8)	95(5.4)	134(8.4)	69(6.5)
Educational status					< 0.001
Primary school or below	3383(49.5)	1065(44.1)	885(50.2)	843(52.6)	590(55.8)
Middle school	2844(41.6)	1140(47.2)	730(41.4)	606(37.8)	368(34.8)
High school or above	610(8.9)	208(8.6)	149(8.4)	154(9.6)	99(9.4)

Abbreviations: *TC* total cholesterol, *TG* triglyceride, *HDL* high-density lipoprotein, *LDL* low-density lipoprotein, *MetS* metabolic syndrome. * vs. MetS (−) + HHcy (−), P < 0.05; ^#^ vs. MetS (−) + HHcy (+), P < 0.05; ^ vs. MetS (+) + HHcy (−), *P* < 0.05;

In the hyperhomocysteinemia group, the rates of smoking and drinking were relatively higher compared with those in the rest of the groups, whereas the proportion of subjects with higher physical activity decreased in the normal group. Participants who had MetS alone had an increased rate of elevated fast glucose levels, diabetes and abdominal obesity compared with those in the reference group. The group with the coexistence of HHcy and MetS had significantly higher proportions of elevated triglyceride levels, LDL-C and TC and exhibited a higher prevalence of high total cholesterol, high triglyceride, high LDL-C, low HDL-C and low hyperuricemia than those in the reference group.

**Fig. 1:**
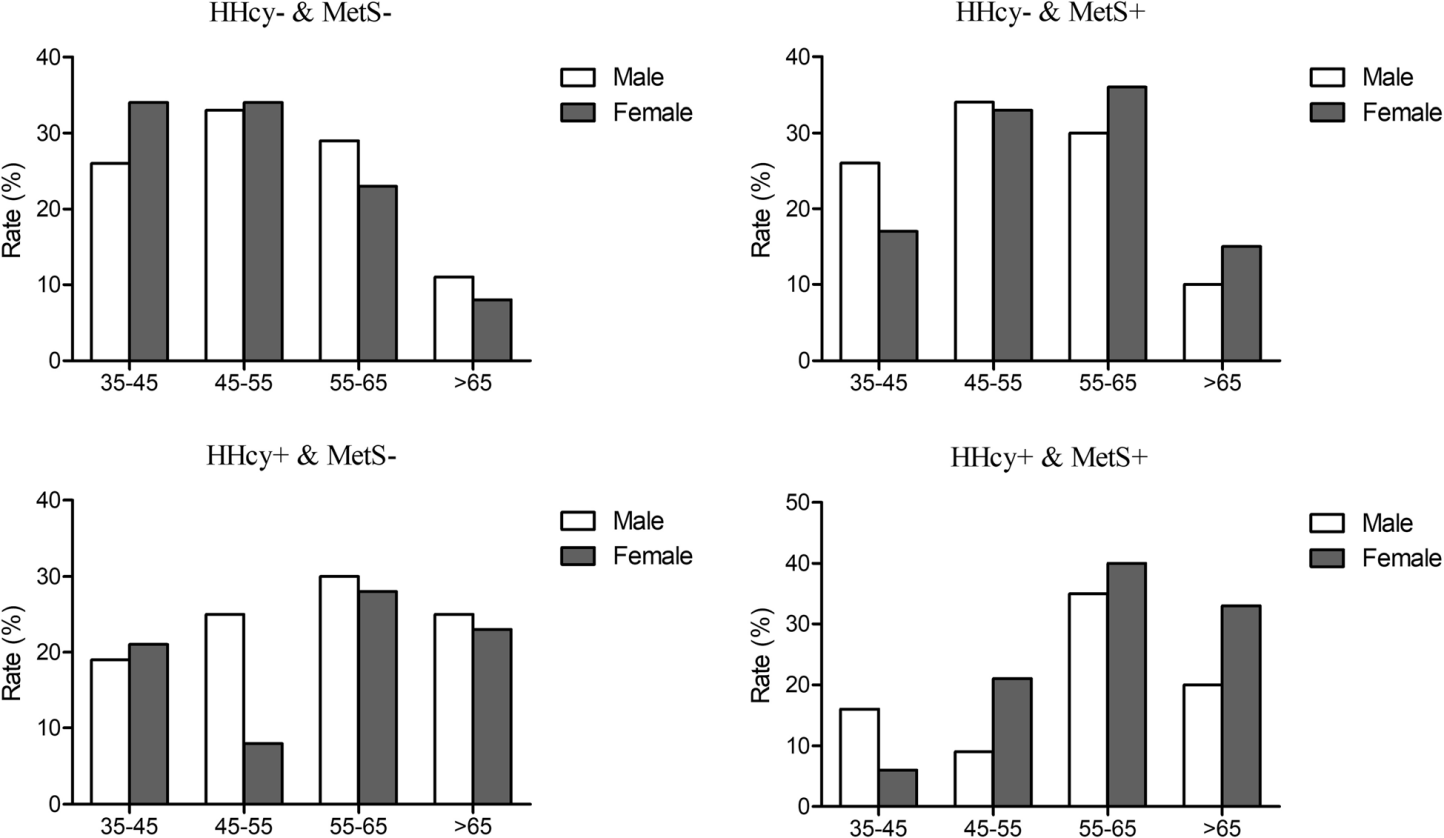
The prevalence of different subgroups of HHcy and MetS according to gender and age groups

Figure 1. shows that in the HHcy alone group and the HHcy and MetS group, the highest prevalence of LVH was in the 55–65 age group in both sexes (30.2% for males; 27.7% for females vs. 35.4% for males; 40.4% for females). For the MetS alone group, the highest prevalences were in the 45–55 age group in males (33.9%) and in the 55–65 age group in females (35.8%). Males with HHcy alone and females with MetS alone had the lowest prevalence in the 35–45 age group (19.4, 5.9%). Similarly, females with HHcy alone and males with MetS alone had the lowest prevalence in the 45–55 age group (7.8, 8.6%). In the MetS alone group, both males and females had the lowest rate in the > 65 age group (10.2, 14.7%).

**Fig. 2:**
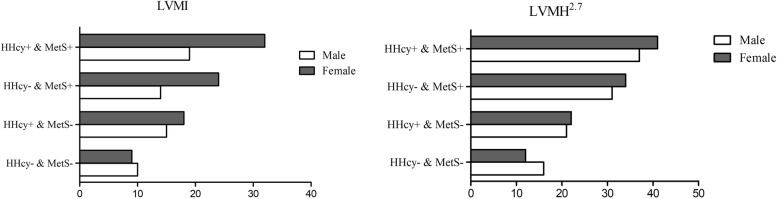
The prevalence of LVH in different gender and different subgroups with or without HHcy or MetS according to different diagnostic criteria

The prevalence of LVH in the total subgroups is shown in Fig. 2. The highest proportion of LVH in males (19.1% for LVMI; 31.6% for LVMH^2.7^) and females (37.3% for LVMI; 40.6% for LVMH^2.7^) was in the different subgroups with coexistent HHcy and MetS, showing a descending trend in females in the subgroup with MetS alone (23.5% for LVMI; 34.0% for LVMH^2.7^) and the subgroup with HHcy alone (17.5% for LVMI; 21.5% for LVMH^2.7^). In males, the rate of LVH increased in the HHcy alone group (14.8%) compared with that in the MetS alone group (13.9%) according to the definition of LVMI, and when using the LVMH^2.7^ criteria, the results were the opposite (21.0% vs. 30.7%).

The cardiac indexes of the study participants were subdivided by the presence or absence of HHcy or MetS, as shown in Table 2. The data show that the highest values of all the cardiac indexes were found in the group with coexistent HHcy and MetS (all *P* < 0.001). Except for the end-diastolic LV internal dimension and LV mass indexed for BSA, all of the mean values gradually increased according to the following sequence: the HHcy alone group, the MetS alone group and the coexistent HHcy and MetS group.

**Table 2:** Cardiac parameters of the study population subdivided according to the presence or the absence of Metabolic syndrome and Hyperhomocysteinemia

	MetS (−)		MetS (+)	
	HHcy (−)	HHcy (+)	HHcy (−)	HHcy (+)
End-diastolic LV internal dimension(mm)	4.61 ± 0.41	4.74 ± 0.43*	4.72 ± 0.42*	4.85 ± 0.44*^#^^
End-diastolic posterior wall thickness(mm)	0.83 ± 0.08	0.86 ± 0.11*	0.88 ± 0.12*^#^	0.90 ± 0.11*^#^^
End-diastolic interventricular septum(mm)	0.85 ± 0.11	0.89 ± 0.15*	0.90 ± 0.12*^#^	0.94 ± 0.14*^#^^
LV mass(g)	144.60 ± 38.59	162.44 ± 47.21*	165.74 ± 47.11*^#^	181.69 ± 50.27*^#^^
LV mass indexed for BSA(gm^−2^)	90.93 ± 22.27	99.19 ± 26.93*	98.77 ± 26.92*	105.01 ± 28.06*^#^^
LV mass indexed for height^2.7^(gm^-2.7^)	40.96 ± 10.93	43.78 ± 13.10*	47.96 ± 13.94*^#^	49.77 ± 14.21*^#^^
Ejection fraction	63.39 ± 3.98	62.667 ± 4.21*	63.16 ± 3.91^#^	62.54 ± 4.42*^^^

Abbreviations: *LV* left ventricular, *MetS* metabolic syndrome. * vs. MetS (−) + HHcy (−), *P* < 0.05; ^#^ vs. MetS (−) + HHcy (+), *P* < 0.05; ^ vs. MetS (+) + HHcy (−), *P* < 0.05;

Table 3 shows the effect of the coexistence of HHcy and MetS on LVH with the crude and adjusted ORs and 95% CIs. The risk of LVH increased in the groups with HHcy (OR: 1.175, 95% CI: 1.002–1.377) and with MetS (OR: 1.615, 95% CI: 1.393–1.873) and showed the highest risk in the coexistent HHcy and MetS group (OR: 1.628, 95% CI: 1.364–1.944) after adjusting for all confounders. Similar associations were observed when using LVMH^2.7^ to diagnose LVH. The risk of the presence of LVH was significantly increased in the groups with HHcy (OR: 1.248, 95% CI: 1.044–1.492) and MetS (OR: 2.567, 95% CI: 2.174–3.032) and in the group with coexistent HHcy and MetS (OR: 2.433, 95% CI: 2.019–2.932) compared with that in the reference group. The interaction of HHcy and MetS had the greatest effect on LVH according to the different diagnostic criteria.

**Table 3:** Association between the presence or the absence of Metabolic syndrome and Hyperhomocysteinemia and LVH

	Unadjusted		Model 1		Model 2		Model 3	
LVMI	OR	95%CI	OR	95%CI	OR	95%CI	OR	95%CI
Non-MetS & normal Hcy	ref		ref		ref		ref	
HHcy & Non-MetS	1.208*	1.049–1.391	1.201#	1.025–1.407	1.201#	1.025–1.407	1.175#	1.002–1.377
Normal Hcy & MetS	2.300*	2.006–2.638	1.820*	1.575–2.103	1.817*	1.572–2.100	1.615*	1.393–1.873
MetS & HHcy	2.284*	1.957–2.666	1.929*	1.627–2.286	1.921*	1.620–2.278	1.628*	1.364–1.944
LVMH^2.7^	OR	95%CI	OR	95%CI	OR	95%CI	OR	95%CI
Non-MetS & normal Hcy	ref		ref		ref			
HHcy & Non-MetS	1.760*	1.494–2.074	1.291#	1.082–1.541	1.291#	1.081–1.541	1.248#	1.044–1.492
Normal Hcy & MetS	3.261*	2.787–3.815	2.974*	2.528–3.499	2.951*	2.508–3.473	2.567*	2.174–3.032
MetS & HHcy	4.143*	3.492–4.915	3.013*	2.519–3.603	2.960*	1.084–3.542	2.433*	2.019–2.932

Model1 adjusted for gender, age, current smoking, current drinking, activity, education

Model2 adjusted for gender, age, current smoking, current drinking, activity, education, high LDL-C

Model3 adjusted for gender, age, current smoking, current drinking, activity, education, high LDL-C, hyperuricemia and medication treatment of hypertension or dyslipidemia

^*^ means *P* < 0.001,^#^ means *P* < 0.05

Abbreviations: *LVH* left ventricular hypertrophy, *MetS* metabolic syndrome

## Discussion

In the present study, we found that HHcy alone was associated with a higher risk of LVH in rural Northeast China. We reconfirmed that MetS alone also increased the risk of LVH. We observed the greatest interaction effect of the coexistent HHcy and MetS group on LVH compared with those of the non-HHcy and non-MetS group and the HHcy or MetS alone group. Therefore, the results from the present study support the hypothesis that HHcy may increase the risk of developing LVH among MetS residents.

The prevalence of HHcy in the present study was 41.3% in the general population. This prevalence was significantly higher than the overall pooled prevalence in China [21]. Although studies have claimed that the prevalence of HHcy is higher in northern areas, the results from our study showed an even higher rate than previously reported. In addition, the prevalence was higher than those of many other places in the world, such as Brazil [22], Lebanon [23], Korea [24] and West Africa [25]. One of the major possible reasons for the high prevalence of HHcy might be the different dietary habits in rural Northeast China. In rural Northeast China, especially during the winter season, pickled cabbage is the mostly commonly consumed vegetable because it is easy to store and does not easily deteriorate [8]. Since the pickling method destroys folate and vitamin B12 in vegetables, it ultimately results in HHcy. Another reason that could possibly explain the higher prevalence of HHcy might be the relatively higher alcohol consumption in the HHcy group than that in the other groups. As shown in Table 1, residents with HHcy with and without metabolic syndrome had higher alcohol consumption than that of residents without hyperhomocysteinemia. Choi and colleagues reported that hyperhomocysteinemia is related to heavy alcohol consumption and low serum levels of folate and vitamin B12 in patients who have had a stroke [26]. Similarly, Coppola confirmed that the plasma homocysteine concentration is associated with craving hazardous and harmful patterns of alcohol consumption [27]. Previously, studies have suggested a relationship between homocysteine and LVH. Kharlamova and colleagues claimed that a positive relationship was found between the concentrations of homocysteine and LV mass (LVM), which suggests that homocysteine has an unfavorable effect on the structure and function of LV [28]. Peer and colleagues also determined that homocysteine, as well as CRP, was significantly positively associated with LVM and LVMI in females [29]. Many previous studies have confirmed the possible relationship between homocysteine and LVH. However, all of the previous studies enrolled subjects with programmed hemodialysis or hypertension, and the coexistence of other conditions or diseases may change the effect of homocysteine on LVH. In the Framingham Heart Study, data from female participants who did not have heart failure or a previous myocardial infarction had homocysteine levels that were directly related to left ventricular mass and wall thickness [28]. In our present study, we also found that HHcy alone could increase the risk of LVH in the general population of rural Northeast Chinese residents, which agrees with many other previous studies. The possible reasons for the hyperhomocysteinemia-induced cardiac hypertrophy might be the increase in oxidative stress and density of mast cells caused by hyperhomocysteinemia in the heart [30] or the activation of protein kinase C [31] and alteration of collagen metabolism [32]. In the present study, we demonstrated that even HHcy alone increased the risk of LVH. Our research team previously reported that higher plasma homocysteine levels were associated with a long QTc interval [33]. A long QTc interval is relevant to ventricular arrhythmia, which might result in sudden cardiac death [34]. Until now, the mechanism of Hcy-mediated cardiac arrhythmias has remained unclear, but our study determined that HHcy increases the risk of LVH and is associated with ventricular arrhythmias. Therefore, it might be possible that HHcy mediates LVH and causes a long QTc [35].

As far as we know, there is limited research on the possible effects of the coexistence of HHcy and MetS on LVH using different sonographic clinical indicators. Studies have aimed to determine the relationship between HHcy, MetS and LVH separately [36, 37]. To the best of our knowledge, the present study is the first to evaluate the combined effect of HHcy and MetS on LVH. The results show that both HHcy alone and MetS alone increase the risk of LVH in the general population from rural Northeast China. Furthermore, the combination of HHcy and MetS lead to greatest risk of LVH. Previous studies have confirmed that H type hypertension, which is characterized by a high level of homocysteine, is associated with a higher risk of stroke [38, 39]. Therefore, we treated patients who were targeted to decrease the level of homocysteine. In the present study, we reported that, in the general population, HHcy alone can increase the risk of LVH and the coexistence of HHcy and MetS can further increase this risk. Therefore, we should place more emphasis on the role of homocysteine. A routine body check should include a homocysteine test, and patients who have been diagnosed with MetS should check their homocysteine levels more frequently to prevent the development of LVH. Furthermore, we should not only focus on the treatment of metabolic disorders but also realize that reducing HHcy is important.

There are some limitations to our investigation. First, no causal relationship was able to be determined from the present study due to the innate drawbacks of cross-sectional studies. Second, we did not evaluate whether the participants had ever received a folic acid fortification treatment. Third, a selection bias may exist because there some participants were excluded due to a lack of laboratory assessments and ultrasonic data.

## Conclusion

The results of this community-based population study demonstrate that HHcy has either an independent or a combined effect with MetS on the presence of LVH.

## Data Availability

Enquiries regarding the availability of primary data should be directed to the principal investigator Professor Yingxian Sun (sunyingxian12@aliyun.com).

## Abbreviations

BMI: Body mass index
BP: Blood pressure
CAD: Coronary artery disease
EDTA: Ethylenediaminetetraacetic acid
FPG: Fasting plasma glucose
HDL: High-density lipoprotein cholesterol
HHcy: Hyperhomocysteinemia
LDL: Low-density lipoprotein cholesterol
LV: Left ventricular
LVH: Left ventricular hypertrophy
LVM: LV mass
LVMH: Left ventricular mass index for height
LVMI: Left ventricular mass index
MetS: Metabolic syndrome
OSAS: Obstructive sleep apnea syndrome
TC: Total cholesterol
TG: Triglycerides
WC: Waist circumference
